# Intractable Delayed Bleeding After Endoscopic Submucosal Dissection for Early Gastric Cancer in Patients With Chronic Disseminated Intravascular Coagulation Caused by Aortic Aneurysm

**DOI:** 10.1002/deo2.70277

**Published:** 2026-01-30

**Authors:** Hiroyuki Endo, Waku Hatta, Noriyuki Obara, Kasumi Hishinuma, Tomoyuki Koike, Atsushi Masamune

**Affiliations:** ^1^ Department of Gastroenterology Japan Community Health Care Organization Sendai Hospital Japan; ^2^ Division of Gastroenterology Tohoku University Graduate School of Medicine Japan

**Keywords:** aortic aneurysm, chronic disseminated intravascular coagulation, endoscopic submucosal dissection, intractable bleeding, stomach

## Abstract

Chronic disseminated intravascular coagulation (DIC) is a rare complication of an aortic aneurysm (AA), and it may go unnoticed because patients are often asymptomatic. The condition is sometimes first recognized when trauma or an invasive procedure triggers a sudden and severe difficulty in achieving hemostasis. Here, we report a case of chronic DIC that was diagnosed following intractable delayed bleeding after endoscopic submucosal dissection (ESD) for early gastric cancer. The patient underwent two ESD procedures, one in 2021 and another in 2023, and experienced delayed bleeding after both. In 2021, hemostasis was easily achieved, and complication of his hemodialysis was suspected as the cause of subsequent delayed bleeding. However, when hemostasis proved difficult in 2023, chronic DIC caused by an AA was identified as the primary cause of the intractable bleeding. Although the patient had a mildly reduced platelet count before the initial ESD, the presence of chronic DIC went unnoticed. The successful hemostasis during the first procedure obscured the underlying cause of the bleeding and thrombocytopenia. Gastroenterologists should be aware of enhanced‐fibrinolytic‐type DIC associated with an AA and remain vigilant regarding its high bleeding risk when performing invasive treatments, including endoscopic procedures.

**Abbreviations**
AA: aortic aneurysm
CT: computed tomography
DIC: disseminated intravascular coagulation
EGD: esophagogastroduodenoscopy
ESD: endoscopic submucosal dissection
FFP: fresh frozen plasma
POD: postoperative day.

## 1 Introduction

Chronic disseminated intravascular coagulation (DIC) is a rare but serious complication of an aortic aneurysm (AA) [1]. The condition often goes unnoticed because patients may remain asymptomatic despite clear evidence of coagulation‐fibrinolytic system activation [2]. Persisting bleeding following trauma or invasive procedures, such as dental extractions, has been reported as a diagnostic clue for chronic DIC [3]. However, reports involving endoscopic procedures are extremely limited. Here, we report a case of chronic DIC diagnosed based on intractable delayed bleeding after endoscopic submucosal dissection (ESD) for early gastric cancer.

## 2 Case Report

**FIGURE 1:**
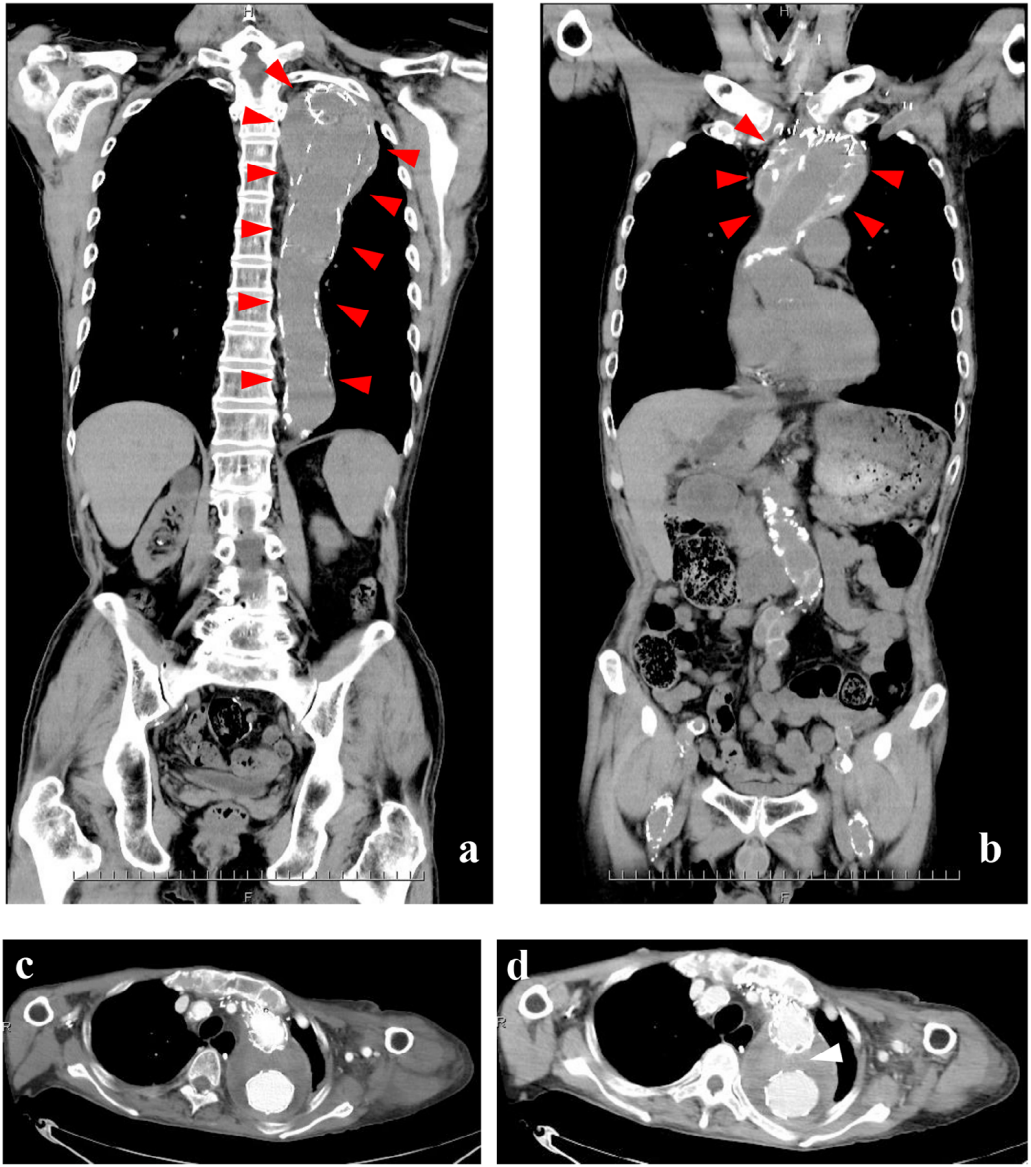
The CT findings of an aortic aneurysm. Abdominal (a) and thoracic (b) aortic aneurysms were replaced with vascular grafts in 2013 and 2019, respectively. The endoleak of aortic aneurysm was revealed by enhanced CT scan on POD 29 in 2023 (c, d, white arrow). CT, computed tomography; POD, postoperative day.

**FIGURE 2:**
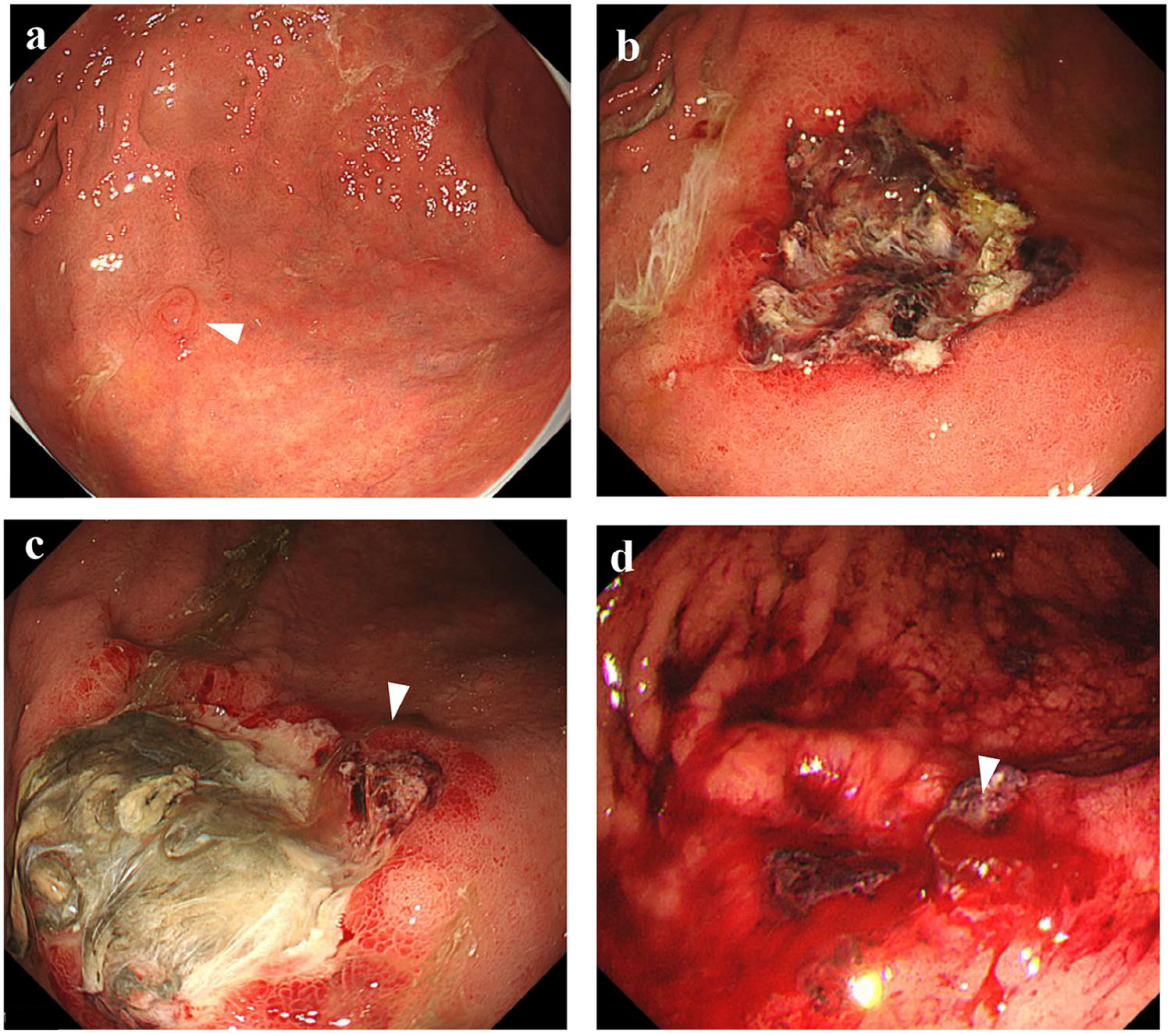
Endoscopic images from the perioperative period of gastric ESD in 2021. A 7‐mm‐diameter, slightly elevated lesion located at the greater curve of the middle body (white arrow) (a) was resected by ESD without severe bleeding, and the second‐look EGD on POD 1 did not detect any hemorrhage (b). The third‐look EGD on POD 8 revealed a viable vessel (white arrow) (c) of ESD ulcer, and it was preventively coagulated. However, delayed bleeding from the vessel that coagulated on POD 8 occurred on POD 14 (white arrow) (d), and additional coagulation was needed. EGD, esophagogastroduodenoscopy; ESD, endoscopic submucosal dissection; POD, postoperative day.

In 2021, a 70‐year‐old Japanese man was hospitalized for the treatment of early gastric cancer. He had a significant history of AA, having undergone two vascular graft surgeries at another medical institution (Figure 1). Because of severe comorbidities, including chronic kidney disease requiring hemodialysis, his gradually enlarging aortic arch and abdominal aneurysm were managed conservatively rather than surgically. His laboratory data on admission are shown in Table 1. The patient's platelet count was below the normal range, a finding that a gastroenterologist at the previous medical institution had attributed to liver cirrhosis. Although the patient's liver computed tomography (CT) image did not clearly demonstrate cirrhosis, we accepted the diagnosis because no alternative explanation was identified, and cirrhosis could not be completely ruled out. The patient was not taking any antithrombotic drugs. Esophagogastroduodenoscopy (EGD) revealed a 7‐mm, discolored, slightly elevated lesion on the greater curvature of the middle body (Figure 2a), which was successfully resected by ESD. On postoperative day (POD) 1, second‐look EGD showed neither hemorrhage nor viable vessels at the resection site (Figure 2b). However, a viable vessel was detected during third‐look EGD on POD 8, prompting prophylactic coagulation (Figure 2c). The patient was discharged on POD 9 but experienced delayed bleeding on POD 14 from the previously coagulated site (Figure 2d). Hemostasis was achieved by electrocoagulation, and no further re‐bleeding occurred. Although laboratory data on POD 14 revealed hemostatic abnormalities (Table 1), no further investigation was pursued because complete hemostasis had been achieved. At the time, the patient's had a BEST‐J score [4] of 3 points due to his hemodialysis, which was suspected to be the main cause of the delayed bleeding.

**TABLE 1:** Changes in laboratory data.

	2013		2019		2021		2023	
	Before surgery	2016	Before surgery	2020	Before ESD	POD 14	Before ESD	POD 10
White blood cell (/µL)	5960	4840	4070	5110	4300	5310	3930	4550
Red blood cell (×10^4^/µL)	422	315	266	290	380	239	355	269
Hemoglobin (g/dL)	13.2	9.9	8.3	8.6	11.8	7.9	10.6	8.1
Platelet (×10^4^/µL)	13.3	11.0	11.2	12.6	8.1	5.9	8.5	7.4
PT‐INR	1.10	1.12	1.01	1.06	1.15	1.29	1.19	1.62
FDP (µg/mL)	−	−	−	−	−	87.9	−	65.5
Fibrinogen (mg/dL)	298	186	228	−	−	135	−	99
D‐dimer (µg/mL)	46.6	24.9	28.1	76.8	−	33.7	−	26.1

Abbreviations: ESD, endoscopic submucosal dissection; FDP, fibrinogen degradation products; POD, postoperative day; PT‐INR, prothrombin time international normalized ratio.

**FIGURE 3:**
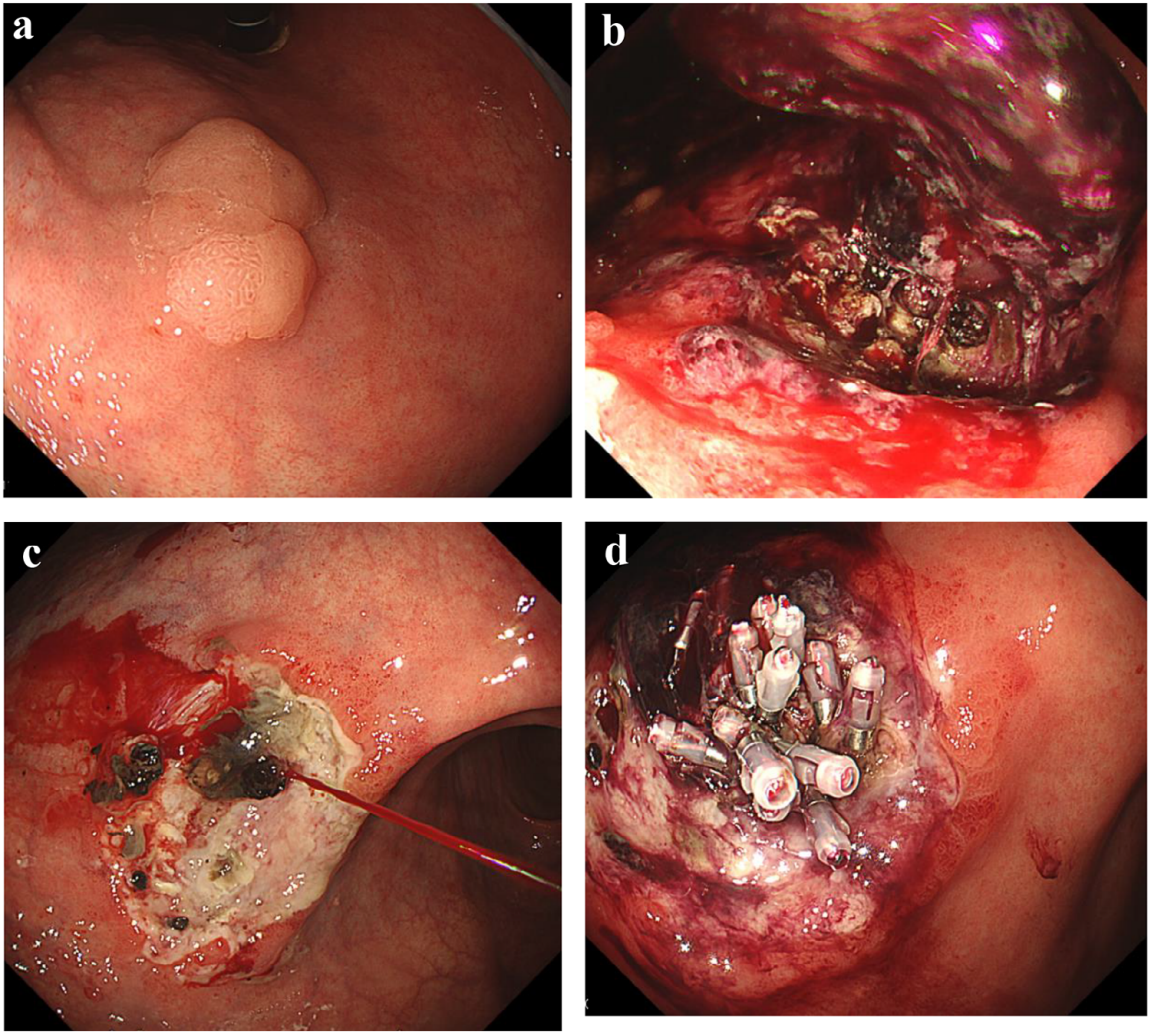
Endoscopic images from the perioperative period of gastric ESD in 2023. ESD was done for a 10‐mm‐diameter, elevated lesion located at the lesser curve of the lower body (a), and frequent bleeding occurred during the procedure, and coagulation was needed for each bleeding. Additional hemostasis was needed for oozing bleeding of the ESD ulcer on the second‐look EGD on POD 1 (b). Spurting bleeding occurred during the third‐look EGD on POD 10 (c), and hemostasis could be achieved by clipping with many difficulties (d). ESD, endoscopic submucosal dissection; EGD, esophagogastroduodenoscopy; POD, postoperative day.

In 2023, the patient underwent ESD for a metachronous early gastric cancer. The lesion was a 10‐mm, discolored, elevated mass located on the lesser curvature of the lower body (Figure 3a). On admission, his laboratory data were nearly identical to those from 2021 (Table 1). Notably, frequent bleeding occurred during the procedure, which was controlled with electrocoagulation. Additional hemostasis was required for oozing bleeding from the ESD ulcer during the second‐look EGD on POD 1 (Figure 3b). However, during the third‐look EGD on POD 10, spurting bleeding occurred and proved intractable as it could not be controlled with electrocoagulation (Figure 3c). Hemostasis was eventually achieved, though with great difficulty, by applying additional clips (Figure 3d). On POD 11, a Mallory‐Weiss tear, likely caused by the prolonged endoscopic treatment performed the previous day, resulted in new bleeding (Supporting Information 1a), necessitating further hemostasis. Although this bleeding was successfully controlled with electrocoagulation, the patient immediately complained of severe abdominal pain. A subsequent CT scan revealed free intraperitoneal air (Supporting Information 1b), confirming perforation of the upper gastrointestinal tract. We suspected that repeated coagulation attempts on POD 10, along with carbon dioxide insufflation during endoscopic hemostasis on POD 11, had weakened the ESD ulcer and led to the perforation. Emergent surgery was performed, which confirmed a perforation at the ESD ulcer site (Supporting Information 1c). To control the perforation and hemorrhage, a partial gastrectomy, including the ESD ulcer site, was performed. However, re‐bleeding from a Mallory‐Weiss tear occurred the very next day, once again requiring endoscopic hemostasis. This persistent bleeding prompted consideration of a cause for the coagulopathy beyond the patient's hemodialysis. The blood test on POD 10 revealed hemostatic abnormalities similar to those observed in 2021 (Table 1). When emergent surgery was considered, a vascular surgeon familiar with enhanced‐fibrinolytic‐type DIC associated with AA recognized its presence, based on the hemostatic abnormalities, which scored 10 points according to the criteria of the Japanese Ministry of Health and Welfare [5]. Dynamic CT findings on POD 29, which revealed an endoleak of the AA, were also consistent with this diagnosis (Figure 1c,d). Following the diagnosis, the patient was treated with transfusions of platelets and fresh frozen plasma, and no further hemorrhagic complications occurred since POD 13. Unfortunately, his condition worsened when he developed paralytic ileus as a perioperative complication on POD 18, which led to aspiration pneumonia. Despite intensive care, he died on POD 31. A timeline diagram illustrating the clinical course is provided (Supporting Information 2).

## 3 Discussion

To our knowledge, this is the first report of intractable delayed bleeding after ESD in a patient with chronic DIC caused by an AA. In addition to the previously reported case related to cold snare polypectomy [6], our case further suggests a substantial risk of hemorrhagic complications during any endoscopic procedure in such patients. Notably, our case highlights the severity of the risk, as repeated hemostatic interventions for intractable bleeding led to gastrointestinal perforation, ultimately initiating a cascade that resulted in the patient's death.

As reported in the medical literature, chronic DIC often goes unnoticed due to its asymptomatic nature, with persistent bleeding after an invasive procedure serving as a key diagnostic clue [2, 3]. Although hemostatic abnormalities were noted on POD 14 during the initial ESD in 2021, the successful achievement of complete hemostasis for the delayed bleeding led us to overlook their underlying cause. Hemodialysis was initially considered a plausible cause of the delayed bleeding. However, it could not fully explain the severe, recurrent bleeding the patient experienced in 2023. A vascular surgeon was the first to identify chronic DIC based on the laboratory data. This marked the first time our team recognized chronic DIC caused by an AA as an underlying factor in the patient's intractable bleeding. This pivotal diagnosis prompted a retrospective review of the patient's previous laboratory data from the prior medical institution (Table 1). The patient's thrombocytopenia and hemostatic abnormalities were noted as early as 2013 and persisted over time, suggesting a long‐standing condition of chronic DIC.

Although the combination of tranexamic acid and heparin has been reported as a therapeutic strategy for enhanced‐fibrinolytic‐type DIC, consultation with a hematologist is recommended because inappropriate use of tranexamic acid can cause fatal systemic thrombosis [7, 8]. We were hesitant to administer tranexamic acid because there were no hematologists available for consultation at our hospital, and we could not ignore the risk of fatal systemic thrombosis. Instead, the anticoagulant used during hemodialysis was changed from dalteparin to nafamostat, and transfusions of platelets and fresh frozen plasma (FFP), which are recommended as replacement therapy in bleeding cases, were administered [7]. In our case, these measures may have contributed to managing the patient's hemorrhagic complications, as no such complications were observed after POD13.

In conclusion, gastroenterologists should be aware that an AA can provoke enhanced‐fibrinolytic‐type DIC, a condition that may go unnoticed due to its asymptomatic nature. If a patient with an AA is found to have unexplained thrombocytopenia, hemostatic abnormalities, including fibrinogen, fibrinogen degradation products, and D‐dimer, should be evaluated to determine the possibility of chronic DIC. If such abnormalities are detected, consultation with a hematologist should be considered for further diagnostic and management of chronic DIC. The indication for endoscopic treatments in patients with chronic DIC caused by an AA should be carefully considered, and a strategy for managing potential hemorrhagic complications should be thoroughly discussed in advance. Gastroenterologists must be vigilant about the high risk of bleeding posed by this condition when performing invasive procedures, including endoscopic treatments.

## Author Contributions

**Hiroyuki Endo**: writing – original draft, writing – review and editing, conceptualization, and investigation. **Waku Hatta**: writing – review and editing, and supervision. **Noriyuki Obara**: investigation. **Kasumi Hishinuma**: investigation. **Tomoyuki Koike**: writing – review and editing, and supervision. **Atsushi Masamune**: writing – review and editing, and supervision.

## Conflicts of Interest

Waku Hatta is an Associate Editor of DEN Open. The other authors declare no conflicts of interest.

### Funding

The authors have nothing to report.

### Ethics Statement

All procedures followed have been performed in accordance with the ethical standards laid down in the 1964 Declaration of Helsinki and its later amendments. Ethical approval was not required for this study in accordance with local and national guidelines.

### Consent

Since the patient had passed away, informed consent for publication was obtained from the family of the patient.

## Supporting information




**Supporting Figure S1**: (a) Bleeding (white arrow) was caused by a Mallory‐Weiss tear on POD 11 in 2023. (b) CT detected free intraperitoneal air (red arrow), indicating perforation of the upper gastrointestinal tract. (c) A perforation at the site of the ESD ulcer (white arrow) was confirmed during emergent surgery. CT, computed tomography; ESD, endoscopic submucosal dissection; POD, postoperative day. **Supporting Figure S2**: A timeline diagram that illustrates the clinical course. FFP, fresh frozen plasma; Hb, hemoglobin; PC, platelet concentrates; PLT, platelet; RBC, red blood cells.

## References

[1] Y. Zhang , C. Li , M. Shen , et al., “Aortic Aneurysm and Chronic Disseminated Intravascular Coagulation: A Retrospective Study of 235 Patients,” Frontiers in Medicine 11 (2017): 62–67. 10.1007/s11684-017-0498-7 28233246

[2] Y. Kadohira , S. Yamada , E. Matsuura , et al., “Aortic Aneurysm‐associated Disseminated Intravascular Coagulation That Responded Well to a Switch From warfarin to rivaroxaban,” Internal Medicine 56 (2017): 2913–2917. 28943552 10.2169/internalmedicine.8666-16 PMC5709638

[3] K. A. Peters , P. T. Triolo , and D. L. Darden , “Disseminated Intravascular Coagulopathy: Manifestations After a Routine Dental Extraction,” Oral Surgery, Oral Medicine, Oral Pathology, Oral Radiology, and Endodontics 99 (2005): 419–423. 15772592 10.1016/j.tripleo.2004.08.017

[4] W. Hatta , Y. Tsuji , T. Yoshio , et al., “Prediction Model of Bleeding After Endoscopic Submucosal Dissection for Early Gastric Cancer: BEST‐J Score,” Gut 70 (2021): 476–484. 32499390 10.1136/gutjnl-2019-319926 PMC7873424

[5] N. Kobayashi , T. Maekawa , M. Takada , et al., “Criteria for Diagnosis of DIC Based on the Analysis of Clinical and Laboratory Findings in 345 DIC Patients Collected by the Research Committee on DIC in Japan,” Bibliotheca Haematologica 49 (1983): 265–275. 10.1159/000408467 6667250

[6] H. Iwagami , T. Akamatsu , and Y. Yamashita , “Case of Chronic Disseminated Intravascular Coagulation Associated With Abdominal Aortic Aneurysm With Repeated Bleeding After Cold Snare Polypectomy,” Digestive Endoscopy 34 (2022): 247. 34674392 10.1111/den.14176

[7] H. Asakura , “Diversity of Disseminated Intravascular Coagulation and Selection of Appropriate Treatments,” International Journal of Hematology 113 (2021): 10–14. 33159644 10.1007/s12185-020-03030-5 PMC7648536

[8] E. Asakura , “Diagnosis and Treatment of Disseminated Intravascular Coagulation (DIC),” Journal of the Japan Society of Internal Medicine 109 (2020): 1378–1385. [Article in Japanese], 10.2169/naika.109.1378.

