# Supplementary material for: Intractable Delayed Bleeding After Endoscopic Submucosal Dissection for Early Gastric Cancer in Patients With Chronic Disseminated Intravascular Coagulation Caused by Aortic Aneurysm
Source: DEN Open. 2026 Jan 30;6(1):e70277. doi: 10.1002/deo2.70277 (PMC12857239; doi:10.1002/deo2.70277)
Supplement: Supplementary file 1 — Supporting Figure S1: (a) Bleeding (white arrow) was caused by a Mallory‐Weiss tear on POD 11 in 2023. (b) CT detected free intraperitoneal air (red arrow), indicating perforation of the upper gastrointestinal tract. (c) A perforation at the site of the ESD ulcer (white arrow) was confirmed during emergent surgery. CT, computed tomography; ESD, endoscopic submucosal dissection; POD, postoperative day. Supporting Figure S2: A timeline diagram that illustrates the clinical course. FFP, fresh frozen plasma; Hb, hemoglobin; PC, platelet concentrates; PLT, platelet; RBC, red blood cells. [file DEO2-6-e70277-s001.pptx]

## Slide 1
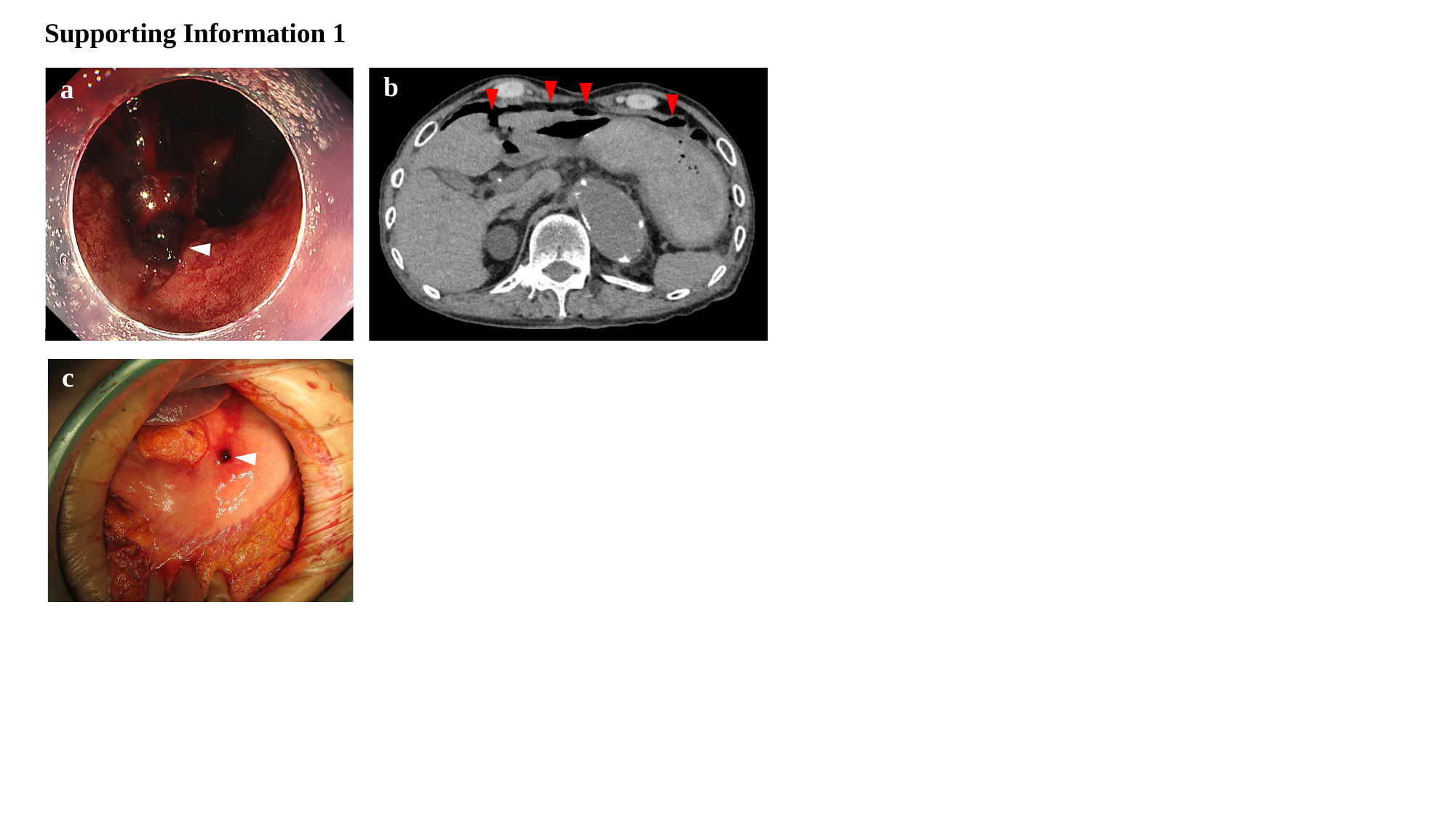

Supporting Information 1
b
a
c

## Slide 2
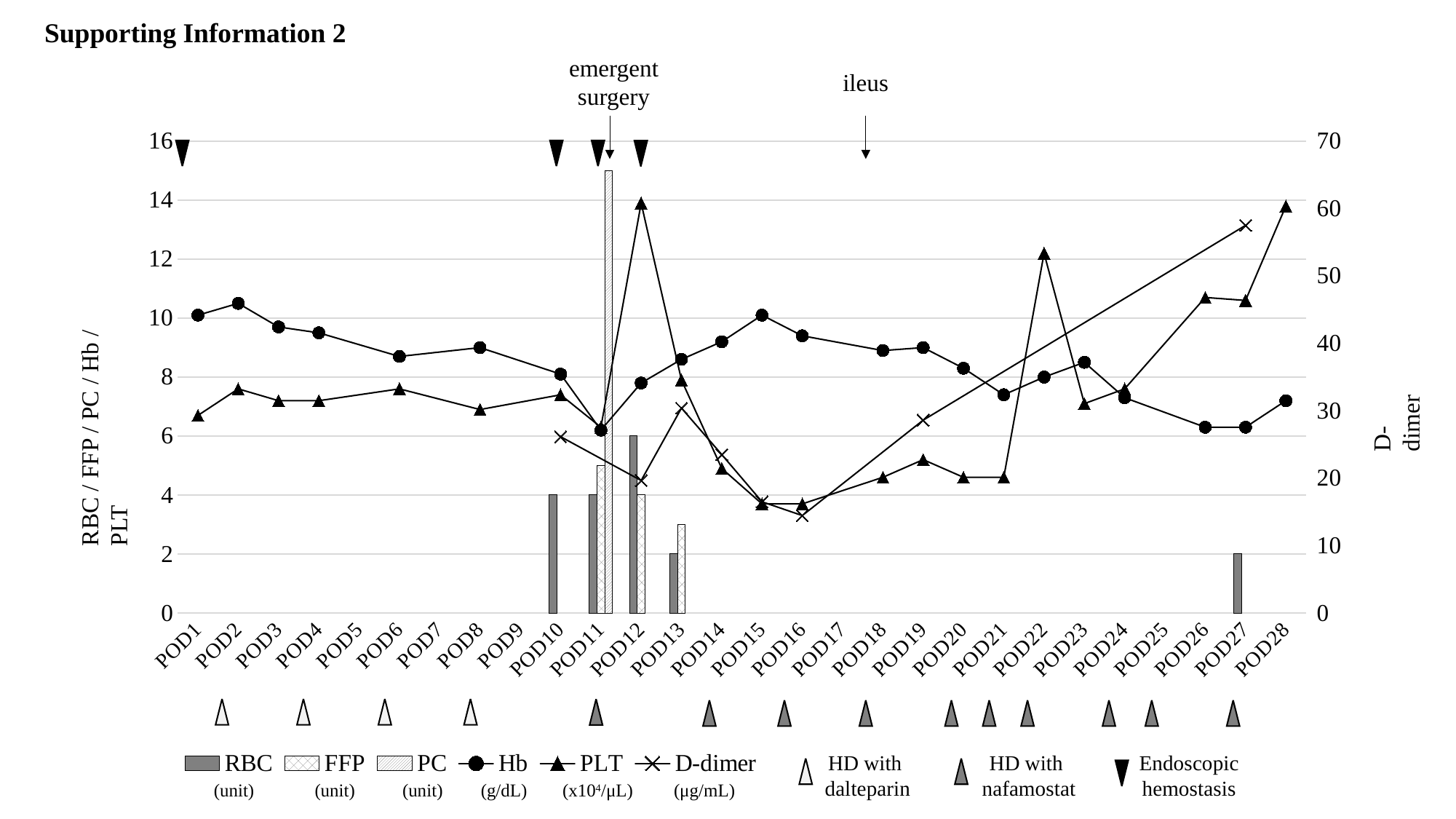

Supporting Information 2
emergent
surgery
ileus
### Chart
| Category | RBC | FFP | PC | Hb | PLT | D-dimer |
|---|---|---|---|---|---|---|
| POD1 | None | None | None | 10.1 | 6.7 | None |
| POD2 | None | None | None | 10.5 | 7.6 | None |
| POD3 | None | None | None | 9.7 | 7.2 | None |
| POD4 | None | None | None | 9.5 | 7.2 | None |
| POD5 | None | None | None | None | None | None |
| POD6 | None | None | None | 8.7 | 7.6 | None |
| POD7 | None | None | None | None | None | None |
| POD8 | None | None | None | 9.0 | 6.9 | None |
| POD9 | None | None | None | None | None | None |
| POD10 | 4.0 | None | None | 8.1 | 7.4 | 26.16 |
| POD11 | 4.0 | 5.0 | 15.0 | 6.2 | 6.3 | None |
| POD12 | 6.0 | 4.0 | None | 7.8 | 13.9 | 19.65 |
| POD13 | 2.0 | 3.0 | None | 8.6 | 7.9 | 30.4 |
| POD14 | None | None | None | 9.2 | 4.9 | 23.5 |
| POD15 | None | None | None | 10.1 | 3.7 | 16.5 |
| POD16 | None | None | None | 9.4 | 3.7 | 14.45 |
| POD17 | None | None | None | None | None | None |
| POD18 | None | None | None | 8.9 | 4.6 | None |
| POD19 | None | None | None | 9.0 | 5.2 | 28.61 |
| POD20 | None | None | None | 8.3 | 4.6 | None |
| POD21 | None | None | None | 7.4 | 4.6 | None |
| POD22 | None | None | None | 8.0 | 12.2 | None |
| POD23 | None | None | None | 8.5 | 7.1 | None |
| POD24 | None | None | None | 7.3 | 7.6 | None |
| POD25 | None | None | None | None | None | None |
| POD26 | None | None | None | 6.3 | 10.7 | None |
| POD27 | 2.0 | None | None | 6.3 | 10.6 | 57.51 |
| POD28 | None | None | None | 7.2 | 13.8 | None |
RBC / FFP / PC / Hb / PLT
D-dimer
HD with
dalteparin
HD with
nafamostat
Endoscopic
hemostasis
(x104/μL)
(μg/mL)
(unit)
(unit)
(g/dL)
(unit)
